# Assessing Autism Co-Occurrence in Fragile X Syndrome: Proposing a Preliminary CARS-2 Cut-Off Score

**DOI:** 10.1007/s10803-025-06888-8

**Published:** 2025-05-21

**Authors:** Tiffany Chavers Edgar, Marianne Elmquist, Claudia Schabes, Latifatu Mohammed, Amy Banasik, Audra Sterling

**Affiliations:** 1 Waisman Center, University of Wisconsin-Madison, 1500 Highland Avenue, Madison, WI, USA; 2 Department of Communication Sciences and Disorders, University of Wisconsin-Madison, Madison, WI, USA

**Keywords:** Fragile x syndrome, Autism, Measurement, Diagnostics

## Abstract

The purpose of this study was to examine the agreement between autism classification from the Autism Diagnostic Observation Schedule (ADOS-2; as reported by Lord (Autism Diagnostic Observation Schedule, Second Edition, Western Psychological Services, 2012)) and the Childhood Autism Rating Scale, 2nd edition (CARS-2; as reported by Schopler (The childhood autism rating scale, second edition (CARS-2), Western Psychological Services, 2010) in individuals with fragile X syndrome (FXS) and to propose a preliminary optimal CARS-2 cut-off score for identifying co-occurring autism in FXS when using the ADOS-2 as gold-standard measure. Forty-three school-aged and adolescent individuals completed the ADOS-2, CARS-2, and cognitive assessments. We found a strong to very strong positive association between the CARS-2 total scores and ADOS-2 total scores for individuals with FXS, and fair agreement between the ADOS-2 and CARS-2 autism classification ratings. Using a receiver-operator characteristic curve, an optimal CARS-2 cut-off score of 24.25 was found for identifying autism in individuals with FXS. Our findings should be considered preliminary, as they represent an examination of measurement agreement rather than validation against comprehensive DSM-5 clinical diagnoses. Studies using CARS-2 in FXS should carefully evaluate the scores or consider using the CARS-2 as a continuous measure of autism traits until the results of this paper are replicated in a larger and more diverse sample.

## Introduction

Fragile X syndrome (FXS) is the leading inherited cause of intellectual disability (Crawford et al., 2001; Verkerk et al., 1991). It is caused by an expansion of the Fragile X Messenger Ribonucleoprotein 1 (*FMR-1*) gene, which is located on the X chromosome. Individuals with FXS have an expanded trinucleotide (CGG) with more than 200 repeats on the *FMR1* gene (Ashley et al., 1993). This leads to an absence or reduction in acceptable levels of the Fragile X Messenger Ribonucleoprotein Protein (FMRP), expressed in neural stem cells (Richter & Zhao, 2021). This suppression or absence of FMRP results in the cognitive, physical, and behavioral phenotypes identified in FXS (Hagerman & Hagerman, 2002). Estimates of the incidence rate of FXS based on genetic testing indicate that 1 in 4000 males and 1 in 7000 females have FXS (Coffee et al., 2009). The phenotypic characteristics of FXS are somewhat more pronounced in males, as almost all females have a second unaffected X chromosome. Specifically, boys with FXS almost universally have cognitive delays and marked deficits in speech and language abilities, including speech intelligibility and phonological inaccuracies (Barnes et al., 2009), expressive and receptive vocabulary (Roberts et al., 2007), morphosyntax (Estigarribia et al., 2011), and pragmatic skills (Klusek et al., 2014a; Martin et al., 2018). In contrast, females with FXS, on average, are less affected, but display a large range of language and cognitive outcomes ranging from intellectual disability and language impairment to average to above average IQs and no language impairment (Kover & Abbeduto, 2019; Mazzocco, 2001; Sterling & Abbeduto, 2012).

FXS is the most common single gene cause of autism (Cohen et al., 2005). Indeed, it is estimated that as many as 90% of males (Abbeduto et al., 2019; Bailey et al., 2001; Haebig et al., 2020; Thurman et al., 2015) and 45% of females with FXS (Hall et al., 2008; Klusek et al., 2014b) demonstrate autistic traits, such as hand flapping, heightened use of perseverative language, repetitive behaviors, and restricted interests. However, some autistic traits observed in FXS may be related to underlying anxiety disorders and/or cognitive impairments (Fielding-Gebhardt et al., 2021). While it is not clear if the behavioral characteristics observed in individuals with FXS reflect the same underlying impairments seen in idiopathic autism, there is agreement that behaviors associated with autistic traits manifest to varying extents in most individuals with FXS (Abbeduto et al., 2019).

Despite most individuals with FXS presenting with some autistic traits, the rates of co-diagnosis of autism in FXS greatly differ across the literature. Specifically, studies have reported that 20–75% of males (e.g., Haebig et al., 2020; Harris et al., 2008) and 3–45% of females with FXS (e.g., Bailey et al., 2009; Caravella & Roberts, 2017; Klusek et al., 2014b) meet the criteria for a co-diagnosis of autism. The large difference in rates of co-occurring autism in FXS may be partly due to differences in autism diagnostic tools. Two commonly used measures in the FXS literature are the Autism Diagnosis Observation Schedule, second edition (ADOS-2; Lord et al., 2012) and the Childhood Autism Rating Scale, second edition (CARS-2; Schopler et al., 2010), which were both designed to identify idiopathic autism (Abbeduto et al., 2019). Therefore, there are concerns about the precision of these measures when applied to individuals with FXS, a population in which autism is considered syndromic by definition (Abbeduto et al., 2014, 2019). Accurate diagnoses of autism in individuals with FXS are critically necessary as it may lead to better understanding of the developmental pathways that lead to the diagnosis (Abbeduto et al., 2014). Thus, the present study aimed to compare rates of autism classification between the CARS-2 and ADOS-2 in individuals with FXS and to explore optimal CARS-2 cut-off scores for identifying co-occurring autism in FXS.

## Autism Diagnostic Measures

The Autism Diagnosis Observation Schedule, second edition (ADOS-2; Lord et al., 2012) and the Childhood Autism Rating Scale, second edition (CARS-2; Schopler et al., 2010) are two of the most widely used autism diagnostic measures in FXS. Both measures rely on direct observation to assess autistic traits but differ in their structure and methods of assessment. Studies in both autism (e.g., Chlebowski et al., 2010; Park et al., 2018; Tachimori et al., 2003) and FXS (e.g., Fielding-Gebhardt et al., 2021; Haebig et al., 2020) have compared the ADOS to the CARS, highlighting potential differences in outcomes. Despite these differences, researchers frequently use either the ADOS-2 or CARS-2 (e.g., Martin et al., 2017; Klusek et al., 2014a; Roberts et al., 2018) when examining autism prevalence in FXS, potentially leading to inconsistent prevalence estimates across studies. To date, there is limited research comparing the performance of these measures within the same sample of individuals with FXS. Therefore, our study aims to understand how these widely used measures compare when applied to the same participants with FXS, and whether standard cut-off scores yield comparable information about autism co-occurrence in this population. Examining agreement between these two autism diagnostic measures can enhance the interpretability of findings across studies using the ADOS-2 and CARS-2.

### ADOS-2

The ADOS-2 is considered part of the gold-standard autism diagnostic battery (Lord et al., 2012). It is a semi-structured assessment with five modules categorized by the examinee’s age (toddler to adult) and expressive language abilities, ranging from no speech to complex sentences. The ADOS-2 uses various materials and “presses” to create a “social world” to elicit social communication differences and restrictive and repetitive behaviors from the examinee (Lord et al., 2012). The ADOS-2 has rigorous training criteria to establish and maintain the tool’s validity and reliability (Lord et al., 2012). Further, additional training is required for the ADOS-2 to be administered for research purposes. In terms of scoring, the examiner scores the participant’s quality and nature of the participant’s social interactions, communication skills, and restrictive and repetitive behaviors in real-time during the ADOS-2 assessment session. The ADOS-2 has a sensitivity range of 0.90–1.0, indicating strong performance in detecting autism, and a specificity range of 0.34–0.76, showing variability in correctly identifying individuals without autism. The ADOS-2 has commonly been used as the primary measure of autism in FXS (e.g., Bush et al., 2021; Klusek et al., 2014a, 2014b; Martin et al., 2018). High rates of autism have been reported in FXS based on the ADOS-2 (e.g., Fielding-Gebhardt et al., 2021; García-Nonell et al., 2008; Haebig et al., 2020; Hall et al., 2008; Thurman et al., 2015). For example, Fielding-Gebhardt et al. (2021) found that 71% of the sample of 45 adolescents (mean age in years = 16.89) with FXS met autism criteria on the ADOS-2.

### CARS-2

The CARS-2 involves direct observation of the examinee in conjunction with information collected through other sources, such as caregiver reports and, educational, or medical evaluations. The tool can be used by various professionals, such as pediatricians, speech-language pathologists, social workers, and psychologists, who have training and exposure to autism. However, there is no specific training required to utilize the CARS-2. There are two versions of the CARS-2: a high-functioning form (CARS-HF) and a standard form (CARS-ST). The latter is most often used in FXS as it is intended for individuals with intellectual disability, and thus we will refer to the CARS-ST as the CARS-2 throughout the remainder of this manuscript (e.g., Haebig et al., 2020; Klusek et al., 2015; Komesidou et al., 2017). The CARS-2 is particularly adept at distinguishing between autistic children and those with significant cognitive impairments (Morgan, 1988; Schopler et al., 2010; Teal & Wiebe, 1986). The CARS-2 includes a 15-item rating scale covering several core differences in autism. Unlike the ADOS-2, there are no specific instructions or training on how to use the CARS-2 for research. The CARS-2 has a sensitivity of 0.88 and a specificity of 0.86 (Schopler et al., 2010), which are interpreted as good in medical statistics (Carter et al., 2016). Autism prevalence rates in both FXS (e.g., Fielding-Gebhardt et al., 2021; Haebig et al., 2020) and idiopathic autism (e.g., Chlebowski et al., 2010; Park et al., 2018; Tachimori et al., 2003) tend to be lower when measured with the CARS-2 than when measured with the ADOS-2. Specifically, Haebig et al. (2020) found that 32 of the 33 boys with FXS (mean age = 12.27) met the criteria for autism on the ADOS-2 whereas only 17 of that same group met the criteria for autism on the CARS-2. Additionally, in a sample of 237 autistic children between the ages of 24–145 months, Ji et al. (2023) found that 87.34% of children met the criteria for autism using the CARS-2 and 97.46% of the same group of children met diagnostic criteria for autism using the ADOS-2.

Due to the differences in autism prevalence rates in the CARS-2 and ADOS-2, some have begun exploring optimal CARS-2 cutoff scores in autism and other intellectual and developmental disabilities by using ADOS-2 as the gold standard (Chlebowski et al., 2010; Haebig et al., 2020; Tachimori et al., 2003). For instance, using a receiver-operator characteristic (ROC) curve, Chlebowski et al. (2010) determined an optimal CARS-2 cut-off score of 25.5 yielded optimal sensitivity and specificity for preschool autistic children (0.82 and 0.95, respectively, for four-year-old participants), using the ADOS-2 as the gold standard for diagnosis. Additionally, also using the ADOS-2 as a gold standard measure, Tachimori et al. (2003) found that a cut-off score of 25.5 on the Childhood Autism Rating Scale (CARS; Schopler et al., 1988) resulted in best sensitivity and specificity (0.86 and 0.83, respectively) for distinguishing children with pervasive developmental disorder—not otherwise specified (PDD-NOS; mean age = 6 years, 9 months) from children with intellectual disability without autism (mean age = 6 years, 5 months). Lastly, Haebig et al. (2020) found that using the traditional cutoff score of 30 on the CARS-2 resulted in 16 of 33 (48.48%) boys with FXS meeting the criteria for an autism diagnosis across the Autism Diagnostic Interview-Revised (ADI-R;Rutter et al., 2003), ADOS-2, and CARS-2. However, when applying a lower CARS-2 cut-off score of 25.5 proposed by Tachimori et al. (2003) and Chlebowski et al. (2010), agreement between the ADI-R, ADOS-2, and CARS-2 increased to 69.70% (23 of 33 males with FXS). This finding suggests that traditional CARS-2 cut-off scores developed for idiopathic autism may need to be adapted to the FXS population.

## Current Study

While the ADOS-2 is often used as a gold standard measure in autism research, concerns remain about how diagnostic tools designed for idiopathic autism compare when used in other developmental disabilities such as FXS. To better understand the utility of these measures in FXS, it is critical to assess the agreement between commonly used diagnostic tools in this population. Both the CARS-2 and ADOS-2 are commonly used interchangeably in FXS research (e.g., Bullard et al., 2021; Friedman et al., 2018; Moskowitz et al., 2024; Neal et al., 2022; Richards et al., 2023). However, studies that have established optimal CARS-2 cut-off scores using the ADOS-2 as the gold standard suggest that adjustments to CARS-2 cut-off scores may be necessary for individuals with idiopathic autism (Chlebowski et al., 2010; Mayes et al., 2011) and those with intellectual disability (Tachimori et al., 2003). This raises concerns about whether current CARS-2 cut-off scores are appropriate for individuals with FXS. Therefore, we had three research questions:

1. What is the concurrent validity between CARS-2 total scores and ADOS-2 total scores in individuals with FXS?
  We predicted that there would be a highly positive correlation between CARS-2 total scores and ADOS-2 total scores, based on previous literature documenting a highly positive relationship between CARS-2 and ADOS-2 total scores.
2. Do the rates of autism classification differ between ADOS-2 and CARS-2 in individuals with FXS?
  We predicted that rates of autism classification would be higher on the ADOS-2 compared to the CARS-2, given rates documented in previous studies.
3. What is the preliminary optimal CARS-2 cut-off score for identifying co-occurring autism in FXS when using the ADOS-2 as the gold standard measure?
  We predicted the preliminary optimal cut-off score for identifying co-occurring autism in FXS would be less than 30, based on alternative cut-off scores reported across the autism literature (Chlebowski et al., 2010; Park et al., 2018).

## Methods

### Participants

Participants included 43 individuals with FXS (mean age = 14.03 years; *SD* = 3.58; range: 9.25–22.23 years), drawn from three separate studies. Thirty-eight of the participants were males (mean age = 13.70; *SD:* 3.35; range: 9.25–21.82) and five participants were female (mean age = 16.52 years; *SD:* 4.79; range: 10.56–22.23 years). Participants were recruited via research registries, outreach to FXS organizations, social media, and word of mouth. Three studies contributing data included: one investigating the relationship between language and the brain in boys with FXS and autism, one examining grammatical development in boys with FXS and autism (BLINDED FOR REVIEW), and one assessing expressive language sampling measures in individuals with developmental disabilities across three different timepoints (BLINDED FOR REVIEW). In the latter study, CARS-2 and ADOS-2 data collected at the first time point was utilized in the current study. For all participants, FXS was confirmed by a molecular genetic report prior to the visit. All participants were monolingual, English speakers who communicated with spoken language, ranging from 2–3-word utterances to more complex language. The sample obtained was representative in terms of sex distribution (with a predominance of males) and communication level (Bailey et al., 2009; Wheeler et al., 2021).

The race of the 43 individuals was reported by caregivers as follows: 84% White; 9% African American; 2% Native American and White; and 2% Native American, White, and African American. One participant’s race was not reported. Additionally, 70% of participants were reported to be non-Hispanic, 7% of the participants were reported to be Hispanic, and ethnicity was not reported for 23% of the participants. Participants were included in the current study if they had a full mutation of FXS confirmed by previous genetic testing and completed both the ADOS-2 and CARS-2. See Table 1 for participant characteristics. Parents provided written informed consent, and children provided verbal or written assent. Study procedures were approved by the institutional review board at (BLINDED FOR REVIEW).

### Measures

#### ADOS-2

The ADOS-2 (Lord et al., 2012) is a 40–60-min semi-structured observational assessment from operationally-defined behaviors associated with autism. An examiner who was research-reliable or training to be research-reliable (with a research-reliable examiner present for live scoring and coding) administered the appropriate ADOS-2 module based on the participant’s language level. The research reliable examiner’s scores were used for data. Three participants were administered Module 1 (word-level language), 22 participants were administered the Module 2 (phrase-level language), and 18 participants were administered the Module 3 (verbally fluent). To ensure consistency across all participants, the ADOS-2 was administered at the end of each session. For module 1, overall total scores above 16 for individuals with few to no words or above 12 for individuals with some words were classified as autism. For modules 2 and 3, overall total scores above 9 were associated with autism. The Gotham et al. (2009) autism severity scoring algorithm was used as the measure for autism trait classification. Across all modules, scores between 1 and 2 were indicative of minimal-to-no evidence of autistic traits, scores between 3 and 4 were associated with low evidence of autistic traits, scores between 5 and 7 were reflected moderate evidence of autistic traits, and scores of 9–10 were considered high evidence of autistic traits.

#### CARS-2

The CARS-2, Standard Version (Schopler et al., 2010) is a 15-item rating scale to quantify autistic traits. An examiner rates the child’s different behaviors based on observations and interactions with the child. CARS-2 ratings are made on a scale from 1–4, with lower scores indicating “no or minimal autistic traits” and higher scores indicating “severe” autistic traits. For a classification of autism, the CARS-2 recommended cut-off score of 28 was used for participants younger than 13 years old and a cut-off of 30 for participants older than 13 years old. Using the CARS-2 recommendations, total raw scores were obtained to indicate an autism trait severity group. For the participants younger than 13, CARS-2 total scores between 15 and 29 were associated with minimal-to-no traits of autism, 30–36.5 were considered mild-to-moderate traits of autism, and scores above 37 were considered severe traits of autism. For participants older than 13 years, CARS-2 scores between 15 and 27.5 were considered minimal-to-no traits of autism, 28–34.5 were associated with mild-to-moderate traits of autism, and scores above 35 reflected severe traits of autism. As mentioned above, the CARS-2 has good sensitivity (0.88) and specificity (0.86) and correlates with clinical ratings of other autistic diagnostic measures that have been used with individuals with idiopathic autism. In the current study, the primary examiner (who completed non-verbal IQ testing), with input from the ADOS-2 examiner, completed the CARS-2 based on observations from the entire visit. To reduce potential for bias, the ADOS-2 and CARS-2 were scored immediately after administration to minimize memory effects. This approach is common in the existing the literature, particularly in studies comparing autism diagnostic tools (e.g., Fielding-Gebhardt et al., 2021; Haebig et al., 2020; Ji et al., 2023; Mayes et al., 2012; Ventola et al., 2006; Ward et al., 2016) due to practical and logistical considerations. Both examiners had experience in autism and autism diagnostics. Since two examiners completed the CARS-2, scoring was collaborative. See Table 2 for participant’s diagnostic classification for each measure.

#### Non-verbal IQ

Participants’ non-verbal IQ was measured using either the Leiter International Performance Scale, Revised (Leiter-R; Roid & Miller, 1997) or the Stanford-Binet Intelligence Scales, Fifth Edition (SB-5; Roid, 2003). This was due to the data being drawn from three different studies. Other studies have also combined these measures to assess cognitive abilities in individuals with intellectual and developmental disabilities (e.g., Alenezi et al., 2022; Stephenson et al., 2023). See Table 2 for nonverbal IQ scores for each participant.

##### Leiter International Performance Scale, Revised.

Thirty-three participants were administered the Leiter-R to determine their nonverbal IQ. Participants completed the Brief-IQ battery: Figure Ground, Form Completion, Sequential Order, and Repeated Patterns. The Leiter-R provides norm-referenced scores for individuals between 2 and 20 years of age. Scores were standardized using a mean of 100 and a standard deviation of 15. The Leiter-R has demonstrated high reliability and validity compared to other cognitive assessments (Roid et al., 2003) and has been used in individuals with FXS (e.g., Haebig & Sterling, 2017; Klusek et al., 2014b; Kover et al., 2013; Skinner et al., 2005).

##### Stanford-Binet Intelligence Scales, Fifth Edition.

Ten participants were administered the Stanford-Binet Intelligence Scales, Fifth Edition (SB-5; Roid, 2003) to measure their cognitive abilities. Participants completed the nonverbal IQ subscales. From these subscales, five factors are used to provide estimates of cognitive ability: fluid reasoning, knowledge, quantitative reasoning, visual spatial processing, and working memory. The SB-5 provides norm-referenced scores for individuals between 2 and 85 years of age. Scores were standardized using a mean of 100 and a standard deviation of 15. The SB-5 has been found to have high reliability and validity to other cognitive assessments (Roid et al., 2003). Additionally, the SB-5 has frequently been utilized to measure nonverbal cognitive abilities in individuals with FXS (i.e., Hoffmann et al., 2019; Huddleston et al., 2014; Lewis et al., 2006).

### Data Analysis

All analyses were performed using R programming language (Version 4.3.2; R Core team, 2023). While the ADOS-2 distinguished between autism and autism spectrum disorder diagnosis, in our analyses we collapsed these two categories to reflect the DSM-V. We conducted Pearson’s correlations between CARS-2 total scores and ADOS-2 total scores to answer our first research question. Weighted Cohen’s kappa was used to examine the agreement of autism classification between the CARS-2 and ADOS-2 for research question two. For research question three, we used a receiver-operator characteristic (ROC) curve to determine optimal CARS-2 cut-off scores for an autism diagnosis using the ADOS-2 as the gold standard for an autism diagnosis. ROC curves are a commonly used metric to evaluate the diagnostic performance of two diagnostic tools. We obtained threshold curves, specificity, sensitivity, and the area under the curve (AUC). Higher AUC values indicate better classification performance. To measure the uncertainty of the AUC, we used bootstrapping, using 2000 bootstrap replicates. We smoothed the ROC curve for our figures, given our small sample size. We did not use a smoothed ROC to obtain our threshold coordinates (i.e., sensitivity and specificity values) since they cannot be determined for smoothed ROCs. We used the Youden index to obtain the optimal threshold (i.e., optimal CARS-2 cut-off score). ROC analyses were conducted using pROC (Version 1.18.5, Robin et al., 2011). We ran all analyses with males and females combined and then separately for males only. Given the small number of females in our study (i.e., *n* = 5), we did not run separate analyses for the female-only group. We found similar results between the females and males combined, as well as male-only analyses. Therefore, we present the combined results narratively in the results section below, but our tables and figure present both analyses.

## Results

### Research Question 1: Concurrent Validity

For the combined male and female dataset, we found a strong to very strong positive association between the CARS-2 total scores and ADOS-2 total scores. Furthermore, the correlations between the CARS-2 and ADOS-2 module 1 (*r* = 0.75, *p* = 0.000), module 3 (*r* = 0.70, *p* = 0.001), and all modules combined (*r* = 0.83, *p* = 0.000) were significant. See Table 3 for all correlation analyses.

### Research Question 2: Agreement

Using traditional CARS-2 cut-off scores (i.e., 28 or 30), all participants classified as autistic on the CARS-2 also received an autism classification based on the ADOS-2. Furthermore, more participants (*n* = 34) were classified as autistic using the ADOS-2, compared to the CARS-2 (*n* = 20). Table 4 displays the agreement between the ADOS-2 and CARS-2 autism classification data. When combining males and females, we found fair agreement between the ADOS-2 and CARS-2 autism classification ratings (*k* = 0.37, 67.44%).

### Research Question 3: Optimal Cut-Off Scores

**Fig. 1:**
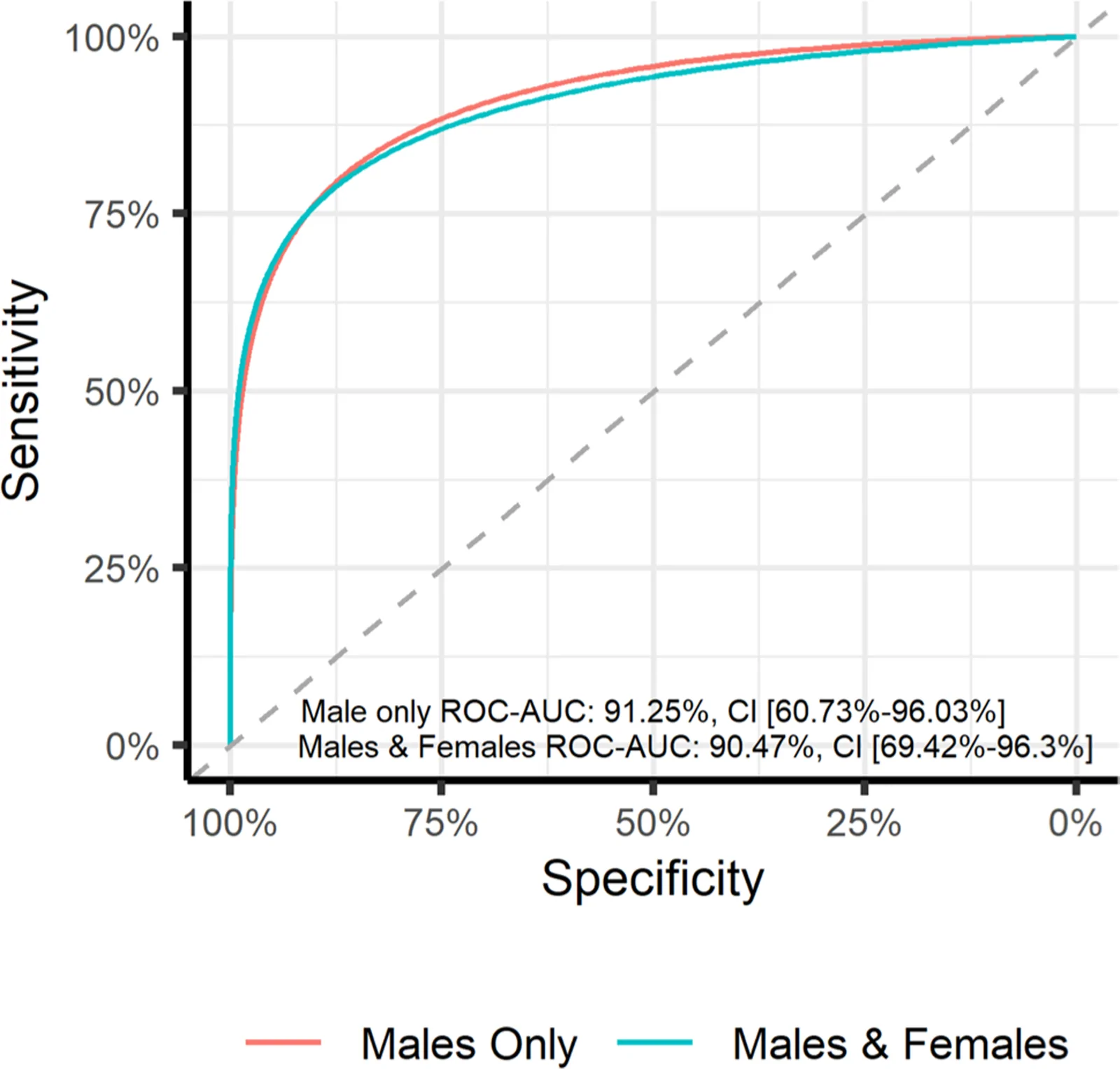
Receiver operator characteristic (ROC) curves showing the performance of the CARS-2 for a co-occurring diagnosis of autism in FXS using the ADOS-2. The red line shows the ROC curve for the males-only analysis, and the blue line shows the ROC curve for the males and females analysis. *ROC-AUC* area under the ROC curve. CI = 95% Confidence intervals. The diagonal dashed line represents the performance of an assessment performing no better than chance

For our third research question, we calculated the area under the curve (AUC) using a ROC graph (See Fig. 1) to examine the diagnostic accuracy of the CARS-2 in identifying co-occurring autism in FXS. Additionally, using the ADOS-2, we also sought to identify a preliminary optimal CARS-2 cut-off score for FXS. For the combined male and female dataset, the bootstrapped AUC for an autism diagnosis on the ADOS-2 was 90.47% (95% CI [72.79, 96.16]). A traditional CARS-2 cut-off score of 30 on the ADOS-2 had 52.94% sensitivity, 100% specificity, 100% positive predictive value, and 36% negative predictive value. We found similar results for a CARS-2 score of 28 (sensitivity = 61.76%, specificity = 100%, positive predictive value = 100%, negative predictive value = 40.91%).

For an autism diagnosis on the ADOS-2, the optimal CARS-2 cut-off score was 24.25 (specificity = 100%; sensitivity = 85.29%, positive predictive value = 100%, negative predictive value = 64.29%). While 24.25 is the optimal CARS-2 cut-off score based on the Youden index, for an autism diagnosis on the ADOS-2, cut-off scores from 22.5 to 25.5 yielded the same sensitivity (i.e., 90.32%) and specificity (i.e., 100%) values as 24.25 (See Table 5).

To evaluate the utility of the preliminary optimal CARS-2 cut-off score for co-occurring autism in FXS, we examined the reliability between the ADOS-2 and CARS-2 diagnostic agreements using the newly derived cut-off score. While the optimal cut-off score based on the Youden index was 24.25, we rounded it down to 24, given that the CARS-2 ratings are based on 0.5 increments, so 24.25 would not be a possible score. Using a cut-off score of 24 on the CARS-2, of the 34 participants classified as autistic on the ADOS-2, 29 were classified as autistic on the CARS-2 (*k* = 0.71, 88.37%; see Table 4). Reliability increased from fair to substantial agreement between the two measures with the revised CARS-2 cut-off score. All participants classified as autistic on the CARS-2, using the newly derived cut-off score of 24, were also classified as autistic on the ADOS-2. While the new preliminary CARS-2 cut-off score increased the number of participants classified as autistic, like the traditional CARS-2 cut-off scores, the ADOS-2 still classified more participants as autistic compared to the CARS-2 (i.e., 34 vs. 29).

## Discussion

The co-occurrence of autism in individuals with FXS has been well-documented, but specific rates have varied across studies (e.g., Bailey et al., 2009; Caravella & Roberts, 2017; Haebig et al., 2020; Harris et al., 2008; Roberts et al., 2018). The varying rates of co-occurring autism and FXS may be attributed to the use of different autism assessment tools across studies (Haebig et al., 2020). Thus, the purpose of this study was to: (1) determine the concurrent validity between CARS-2 total scores and ADOS-2 total scores in individuals with FXS, (2) compare the rates of autism classification between the ADOS-2 and CARS-2 in a sample of 43 individuals with FXS and (3) propose a preliminary CARS-2 cut-off score for identifying co-occurring autism in FXS.

### Agreement Between Autism Diagnostic Tools

In our sample of 43 individuals (38 males, 5 females) with FXS, correlations between the CARS-2 and ADOS-2 total scores were significant. This finding is consistent with previous studies that have reported a significant correlation between the CARS-2 and ADOS-2 in autistic children (Park et al., 2018; Reszka et al., 2014). However, the rates of autism classification differed across the two autism diagnostic measures. Specifically, the ADOS-2 classified the majority (79.06%) of participants with FXS as having co-occurring autism whereas the CARS-2 classified 46.51% of the participants as having co-occurring autism. This finding is remarkable, especially considering that we found a highly positive association between ADOS-2 total scores and CARS-2 total scores. Further, both the CARS-2 and ADOS-2 have high sensitivity and specificity for diagnosing idiopathic autism (i.e., Chlebowski et al., 2010; Park et al., 2018; Ventola et al., 2006).

Previous studies have found high agreement of autism classification between the ADOS and CARS-2 in children with idiopathic autism (i.e., 88–96%; Park et al., 2018; Ventola et al., 2006). Despite our findings regarding ADOS-2 and CARS-2 autism classifications, the classification rates for both assessments fell within the range of rates reported in previous studies involving individuals with FXS. For instance, studies using the ADOS with individuals with FXS have reported rates of co-occurring autism ranging from 13 to 83% (e.g., Clifford et al., 2007; Harris et al., 2008; Klusek et al., 2014b; Thurman et al., 2015) whereas studies using the CARS have reported rates between 20 and 58% of co-occurring autism in individuals with FXS (e.g., Bailey et al., 2001; Demark et al., 2003; Hatton et al., 2006; Klusek et al., 2015).

The lack of agreement between the CARS-2 and ADOS-2 for individuals with FXS may be due to the differences that have been reported for each measure. For instance, though the CARS-2 was updated in 2010 (Schopler et al., 2010), items on the CARS-2 were formulated on autism criteria presented in the DSM-III (American Psychiatric Association, 1980). The test items on the CARS-2 have not been updated since the first edition, which was published in 1988 (Schopler et al., 1988). Schopler et al. (2010) discussed this by stating that the CARS-2 test items have been shown to capture the basic symptoms of autism addressed in all editions of the DSM (American Psychiatric Association,). Recently, the definition of autism has evolved to include a dimensional approach to capture the core autistic traits while allowing for heterogeneity in the quality and quantity of autism symptoms (APA, 2013; Rosen et al., 2021). Additionally, the CARS-2 does not have items focused exclusively on pragmatic language, which is one of the two core domains in the definition of autism. Instead, test items addressing pragmatic language are embedded within test items addressing verbal and non-verbal communication or restrictive and repetitive behaviors. For example, the test item “Adaptation to Change” assesses how the examinee responds to changes in routines or environments, which may overlap with pragmatic difficulties such as adjusting to conversational topics or understanding social change.

Lastly, the inherent structural differences between the ADOS-2 and CARS-2 may impact how autistic traits are measured and subsequently diagnosed in individuals with FXS. For instance, the CARS-2 is a rating scale that measures autistic traits through direct observation of the examinee and information collected through other sources (e.g., parents, therapists) whereas the ADOS-2 is semi-structured assessment that utilizes materials and “presses” to elicit social communication differences and restrictive and repetitive behaviors. Additionally, the CARS-2 uses a single rating scale that can be applied broadly across ages and developmental levels. The ADOS-2, however, has five different modules that are based on the examinee’s verbal abilities and developmental stage. The differences between these measures could lead to potential variations in diagnostic outcomes.

Although the ADOS-2 is considered the “gold standard” diagnostic tool for assessing idiopathic autism, previous studies have questioned the appropriateness of the assessment for diagnosing autism in individuals with FXS. For instance, Abbeduto et al. (2014) suggested that for individuals with FXS, the range of options for scoring items on the ADOS-2 may lead an impairment or unusual behavior to be scored “autistic” simply because it is a better fit than a score reflecting unimpaired or neurotypical. To address this issue, Abbeduto et al. (2014) suggested re-norming of the ADOS-2 for individuals with FXS to allow for finer distinctions to capture the differences between FXS and idiopathic autism. Additionally, Hernandez et al. (2009) and Hall et al. (2010) cautioned administering the ADOS-2 for primary or confirmatory diagnosis of autism in individuals with FXS as it is susceptible for false positives due to the high prevalence of social anxiety in FXS. Indeed, FXS researchers have recommended that adaptations to autism diagnostic tools be made to avoid scores being overly influenced by low nonverbal IQ or behaviors associated with anxiety which are characteristics are FXS (Abbeduto et al., 2014, 2019; Harris et al., 2008; Hogan et al., 2017; Thurman et al., 2015). As there are currently no autism diagnostic tools sensitive to the unique behavioral phenotypes of FXS, diagnostic tools such as the ADOS-2 and CARS-2 should be used with caution and in conjunction with the autism criteria outlined by the DSM-5 (American Psychological Association, 2013).

### Optimal Cut-Off Scores

This preliminary study considered whether the scores provided by the CARS-2 would be optimal cut-off scores for our clinical group. Studies have found varying results, indicating that a score of 30 (or 28 for older children) might not be the most sensitive and specific score for identifying autism. However, those studies were completed with toddlers and/or school age autistic children without FXS (Chlebowski et al., 2010; Park et al., 2018; Tachimori et al., 2003). In all these studies, the authors reported that scores between 25.5–26 yielded better sensitivity and specificity for autism compared to the traditional cut-offs. We found a similar finding for FXS, with our preliminary optimal cut-off score at 24.25. Based on this new preliminary optimal cut-off score, we found substantial agreement between the ADOS-2 and CARS-2 diagnostic categories.

As noted previously, the CARS-2 and ADOS-2 were not designed to measure autistic traits in FXS or other disorders. However, given the rates of autism reported in the literature, and the lack of diagnostic tools available to clinicians and researchers that are normed on FXS, researchers and clinicians have turned to tools like the CARS-2 and ADOS-2 to help describe and understand autism traits in FXS (e.g., Fielding-Gebhardt et al., 2021; Haebig et al., 2020; Hall et al., 2010). However, without a validated score for the CARS-2, comparisons across studies using different diagnostic tools should be carefully considered in interpreting findings. Studies using the CARS-2 in FXS should consider the score carefully or use the CARS-2 as a continuous measure of autistic traits until these findings are replicated in a larger, more diverse sample.

### Study Limitations and Future Directions

Results of this study should be considered given the following limitations. First, we did not have a racially/ethnically diverse sample with most participants identifying as white, non-Hispanic. Given the disparities in diagnosing idiopathic autism among marginalized racial and ethnic groups (e.g., Maenner et al., 2020; Magaña et al., 2013; Pearson et al., 2019), there is a critical need to deepen our understanding of co-occurring autism in FXS among marginalized racial and ethnic individuals.

Second, this study focused on proposing a preliminary optimal CARS-2 cutoff score for identifying co-occurring autism in individuals between the ages of 9–22 with FXS who have spoken communication. Thus, this limits the generalizability of our findings to other FXS profiles (e.g., different ages, language profiles). Additionally, the limited age range prevented us from determining optimal cut-off scores based on specific age groups, as is typically done with CARS-2 cut-off scores (Schopler et al., 2010). Therefore, we identified only a single score for FXS. Future studies should include a wider age range of participants, as well as minimally speaking individuals with FXS, as their autistic traits may differ from our current sample. Third, our sample size was small, especially the number of FXS-only participants. This limits the precision of our ROC statistics and the specificity rates. Additional research with a larger sample that includes more individuals with FXS-only is needed to determine an optimal CARS-2 cut-off score for identifying co-occurring autism in FXS. Although our analyses showed similar results when females were excluded, the limited number of female participants (*n* = 5) highlights the need for larger studies with more females to investigate potential sex differences. Until findings in this paper can be replicated in a larger, more diverse sample, future studies should consider using a more continuous approach to identifying autistic traits in FXS.

Another limitation is that the ADOS-2 and CARS-2 were administered by the same examiners. This dependency could account for some of the overlap between the measures, especially since the CARS-2 is based on a professional’s diagnostic impression of a child and the examiner could use the ADOS-2 to inform their impressions. Although this approach aligns with some clinical and research practices (e.g. Fielding-Gebhardt et al., 2021; Haebig et al., 2020; Ji et al., 2023; Mayes et al., 2012; Ventola et al., 2006; Ward et al., 2016), it may inflate agreement between the diagnostic measures. Additionally, using the same examiners across measures may have facilitated cross-communication, leading to potential bias. Future studies would benefit from independent administration of these measures.

Additionally, inter-rater reliability data was not collected for the scoring of the ADOS-2 and CARS-2. Without inter-rater reliability data, it is unclear to what extent the results reflect consistency across examiners versus individual examiner judgment. This limits the interpretability of our findings and may impact the validity of the preliminary proposed CARS-2 cutoff score. Future research should collect inter-rater reliability data to strengthen the validity and reproducibility of findings.

Lastly, the ADOS-2 was used as the anchoring measure to identify an optimal CARS-2 cutoff score in this study. Although the ADOS-2 is widely considered as one of the gold-standard measures for identifying autistic traits and has strong psychometric properties, it does not constitute a formal diagnosis of autism. A comprehensive autism diagnosis requires clinical judgment based on DSM-5 criteria (American Psychiatric Association, 2013) and ideally incorporates multiple sources of information, such as developmental history, caregiver interviews, and observations across settings. Thus, the absence of a full diagnostic evaluation limits the extent to which our proposed CARS-2 cutoff score can be interpreted as identifying clinically diagnosed autism. However, this study does provide preliminary information that can be used to guide future research focusing on autism diagnostic measures in FXS. Additionally, other measures, such as the ADI-R (Rutter et al., 2003), may be more appropriate for identifying autistic traits in individuals with FXS. Future studies should consider evaluating the utility of additional autism diagnostic tools as well as validate observed autistic traits based on the DSM-5 (American Psychiatric Association, 2013).

## Summary and Conclusions

The present study aimed to further investigate autism diagnosis in individuals with FXS through the administration of the CARS-2 and ADOS-2, and to propose an optimal CARS-2 cut-off score for identifying co-occurring autism in FXS. We observed a strong to very strong positive association between the CARS-2 total scores and ADOS-2 total scores for individuals with FXS, and fair agreement between the ADOS-2 and CARS-2 autism classification ratings. Notably, our results indicated an optimal CARS-2 cut-off score is 24.25 for identifying co-occurring autism in FXS, providing a preliminary benchmark for future research and clinical applications. These results contribute to our understanding and evaluation of autistic traits within the context of FXS. Future research should aim to validate our findings in larger, more diverse sample and evaluate the utility of additional autism diagnostic tools in FXS. Additionally, studies should compare ADOS-2 scores and CARS-2 scores to a DSM consensus diagnosis. Until this study is replicated in a larger and more diverse sample, the CARS-2 should be utilized as a continuous measure of autistic traits.

## Figures and Tables

**Table 1:** Participants demographics

Participant	Mean (SD) n (%)	Range
n	43	
Age	14.03 (3.58)	9.25—22.23 years
Sex- Male	38 (88.37%)	
Ethnicity	3 (7%)	–
Hispanic	30 (70%)	–
Not Hispanic	10 (23%)	
No response		
Race	36 (84%)	–
White	4 (9%)	–
Black or African-American	0 (0%)	–
Native American	1 (2%)	–
Native American; White	1 (2%)	–
Native American; Black or African American; White	1 (2%)	–
No Response		

**Table 2:** Diagnostic classification for each measure and age and nonverbal IQ for each participant

Case No	Gender	Chronological age in years	Nonverbal IQ	CARS-2	ADOS-2
01	M	10;3	61 (Leiter-R)	Autism	Autism
02	M	14;7	43 (Leiter-R)	Autism	Autism
03	M	11;4	49 (Leiter-R)	Autism	Autism
04	M	18;11	36 (Leiter-R)	Autism	Autism
05	M	13;7	45 (Leiter-R)	No autism	Autism
06	M	18;4	34 (Leiter-R)	No autism	Autism
07	M	10;2	43 (Leiter-R)	Autism	Autism
08	M	15;6	55 (Leiter-R)	No autism	Autism
09	M	13;10	36 (Leiter-R)	No autism	Autism
10	M	15;4	34 (Leiter-R)	Autism	Autism
11	M	10;3	36 (Leiter-R)	Autism	Autism
12	M	14;4	40 (Leiter-R)	Autism	Autism
13	M	11;6	36 (Leiter-R)	Autism	Autism
14	M	11;4	40 (Leiter-R)	Autism	Autism
15	M	12;4	42 (Leiter-R)	Autism	Autism
16	M	10;7	52 (Leiter-R)	No autism	Autism
17	M	12;0	36 (Leiter-R)	Autism	Autism
18	M	11;1	44 (Leiter-R)	No autism	Non-spectrum
19	M	14;9	43 (Leiter-R)	No autism	Non-spectrum
20	M	16;0	41 (Leiter-R)	No autism	Non-spectrum
21	M	14;6	72 (Leiter-R)	No autism	Autism
22	M	16;10	36 (Leiter-R)	No autism	Non-spectrum
23	M	17;11	120 (Leiter-R)	No autism	Autism
24	M	13;02	47 (Leiter-R)	No autism	Autism
25	M	9;5	87 (Leiter-R)	No autism	Autism
26	M	9;3	56 (Leiter-R)	No autism	Autism
27	M	9;8	58 (Leiter-R)	Autism	Autism
28	M	13;7	38 (Leiter-R)	No autism	Autism
29	M	14;4	40 (Leiter-R)	No autism	Autism
30	M	11;11	46 (Leiter-R)	Autism	Autism
31	M	11;8	36 (Leiter-R)	Autism	Autism
32	M	13;0	36 (Leiter-R)	Autism	Autism
33	M	9;11	57 (Leiter-R)	No autism	Autism
34	M	21;10	42 (SB-5)	Autism	Autism
35	M	9;4	42 (SB-5)	Autism	Autism
36	F	10;5	42 (SB-5)	Autism	Autism
37	M	17;7	56 (SB-5)	No autism	Non-spectrum
38	M	21;4	42 (SB-5)	Autism	Autism
39	F	17;11	57 (SB-5)	No autism	Non-spectrum
40	F	18;2	44 (SB-5)	No autism	Non-spectrum
41	M	18;6	44 (SB-5)	No autism	Non-spectrum
42	F	12;8	53 (SB-5)	No autism	Non-spectrum
43	F	22;2	46 (SB- 5)	No autism	Autism

*M* male, *F* female, *CARS-2* Childhood Autism Rating Scale, second edition, *ADOS-2* Autism Diagnostic Observation Schedule, second edition, *Leiter- R* Leiter International Performance Scale, Revised, *SB-5* Stanford-Binet Intelligence Scales- fifth edition

**Table 3:** Pearsons product correlations between CARS-2 and ADOS-2 total scores

	ADOS mod 1	ADOS mod 2	ADOS mod 3	ADOS Total
Male and Females CARS-2 Total Score	0.98	0.75**	0.70**	0.83***
Male only CARS-2 Total Score	0.98	0.75***	0.79**	0.84***

*mod* module, *CARS-2* Childhood Autism Rating Scale, 2nd edition, *ADOS-2* Autism Diagnositic Observation Schedule, 2nd Edition

* p < 0.01

** p < 0.001

*** p < 0.000

**Table 4:** Measurement of Agreement (Weighted Cohen’s Kappa)

	κ	95% CI	Interpretation
Males and Females			
Diagnosis (traditional cut-off score)	0.37	0.16, 0.58	Fair
Diagnosis (updated cut-off score)	0.71	0.48, 0.94	Substantial
Males only			
Diagnosis (traditional cut-off score)	0.32	0.10, 0.54	Fair
Diagnosis (updated cut-off score)	0.69	0.41, 0.96	Substantial

*CI* Confidence Interval

**Table 5:** Sensitivity and specificity values for CARS-2 cut-off scores for autism diagnosis on the ADOS-2

CARS-2 cut-off scores	ADOS-2 autism diagnosis			
	Males and Females		Male only	
	Sensitivity %	Specificity %	Sensitivity %	Specificity %
17.5	100	33.33	100	50
18	97.06	44.44	98.88	50
18.5	94.11	44.44	93.75	50
19	91.18	44.44	90.63	50
19.5	91.18	55.56	90.63	66.67
20	91.18	55.56	90.63	66.67
20.5	91.18	55.56	90.63	66.67
21	91.18	66.67	90.63	83.33
21.5	88.24	66.67	87.5	83.33
22	88.24	77.78	87.5	100
22.5	85.29	77.78	87.5	100
23	85.29	88.89	87.5	100
**23.5^a^**	85.29	100	**87.5**	**100**
24	85.29	100	87.5	100
**24.25^b^**	**85.29**	**100**	87.5	100
24.5	85.29	100	87.5	100
25	85.29	100	87.5	100
25.5	85.29	100	87.5	100
26	82.35	100	84.38	100
26.5	79.41	100	81.25	100
27	70.59	100	71.88	100
27.5	64.71	100	65.63	100
28	61.76	100	62.5	100
28.5	55.88	100	56.25	100
29	55.88	100	56.25	100
29.5	55.88	100	56.25	100
30	52.94	100	53.13	100

*CARS-2* Childhood Autism Rating Scale, 2nd edition. *ADOS-2* Autism Diagnostic Observation Schedule, 2nd edition

a Optimal CARS-2 cut-off score for autism diagnosis on the ADOS-2, based on the Youden index for males and females combined

b Optimal CARS-2 cut-off score for autism diagnosis on the ADOS-2, based on the Youden index for males only
